# Book Reviews

**Published:** 1895-11

**Authors:** 


### Book Reviews.

PRACTICAL DIETETICS WITH SPECIAL REFERENCE TO DIET IN DISEASE. By W. GILMAN THOMPSON, M. D., Professor of Materia Medica, Therapeutics and Clinical Medicine in the University of the City of New York; Visiting Physician to the Presbyterian and Bellevue Hospitals, New York. Large 8vo, 800 pages Illustrated. Prices, cloth, $5.00 ; sheep, $6.00. Sold by subscription only. New York: D. Appleton & Co., Publishers, 72 Fifth avenue. 1895.

It is quite true, as asserted by the author of this comprehensive treatise in his introduction, that the subject of the dietetic treatment of disease has not received the attention in medical literature that it deserves. It is equally true that the medical schools have given it inadequate place in their curricula. This work of Thompson will do much to correct both of these evils, and it is hoped that it will stimulate an improvement in the training of nurses in regard to the preparation of food and dietetics, as well as to develop in the hospitals a better order of things in the diet kitchen.

No one is more competent to handle this subject than Gilman Thompson, and it goes without saying that he has done so in an exhaustive manner. The work is as large as most of those on the the theory and practice of medicine thirty years ago, and they sought to cover the whole field of treatment, including dietary management. Thompson begins with the elementary composition of foods and progresses through the entire range of their usefulness and application until he reaches their adaptability to disease. He becomes particularly interesting in the discussion of diseases especially influenced by diet. He does not forget to devote considerable space to the consideration of dietetics in public institutions, and the preparation of food for infants is given adequate place in the book.

It is a work that aims to occupy a field heretofore but little cultivated, and gives evidence of great care in its preparation. It will easily find its way into the library of every thoughtful physician.

THE SCIENCE AND ART OF OBSTETRICS. By THEOPHILUS PARVIN, M. D., LL. D., Professor of Obstetrics and the Diseases of Women and Children in Jefferson Medical College, Philadelphia. New (third) edition. In one 8vo volume of 677 pages, with 267 engravings and two colored plates. Cloth, $4.25; leather, $5.25. Philadelphia: Lea Brothers & Co., Publishers. 1895.

Two previous editions of this work have received extended notices in this journal principally of commendation, hence it will be necessary now only to refer to such changes as the author has made in this edition. These are somewhat briefly stated in the preface to be, first, changes in the order in which the subjects are discussed; second, nearly one-third of the book has been rewritten and, third, additional illustrations have been introduced.

Parvin is a teacher of experience and great literary accomplishment. It is easy to understand, therefore, that his treatise is a popular one. With the principal part of the work there can be no criticism. We are, however, somewhat surprised at the author's position in regard to puerperal fever. Why he has not at once adopted the term puerperal sepsis and discussed the disease from the standpoint that such a name would indicate, we are at a loss to understand. However, much of his pathology is correct and consequently his treatment in the main is satisfactory. We think it would have been well to have discussed phlegmasia alba dolens under the head of puerperal septic phlebitis, a name suggestive of its true pathology and indicating an appropriate line of treatment.

The book as usual is well printed and profusely illustrated. It aims to meet the requirements of both student and obstetrician.

---

THE HISTORY OF PROSTITUTION: Its Extent, Causes and Effects throughout the World. By WILLIAM W. SANGER, M. D., Resident Physician, Blackwell's Island, N. Y. Octavo, pp. 709. Price, cloth, $4.00; leather, $5.00. New York: The American Medical Press, 816 Broadway. 1895.

The subject of this work is not a savory one, yet it is dealt with by the author in a delicate though forceful manner. The solution of the problem has for ages occupied the attention of physicians, humanitarians and legislators. To these we commend a careful study of the work.

The author devoted about seven years to the preparation of the book, basing his observations upon investigations made both in this country and Europe. This is not a book that demands a critical review, but, nevertheless, it merits a patient reading by every person interested in the investigation of the subject. It is one of the most complete works ever issued, and has been brought down to the immediate present through thorough revision. Among those who have contributed to the appendix we notice the names of Drs. F. R. Sturgis, Prince A. Morrow and R. W. Taylor,—names that guarantee the scientific nature of the treatise.

---

A MANUAL OF ORGANIC MATERIA MEDICA; Being a Guide to Materia Medica of the Vegetable and Animal Kingdoms. For the use of Students, Druggists, Pharmacists and Physicians. By JOHN M. MAISCH, Ph. D., Professor of Materia Medica and Botany in the Philadelphia College of Pharmacy. New (sixth) edition, thoroughly revised, by Henry C. C. Maisch, Ph. G. In one 12mo volume of 509 pages, with 285 engravings. Cloth, $3.00. Philadelphia: Lea Brothers & Co., Publishers. 1895.

This celebrated work has heretofore obtained professional favor on both sides of the Atlantic ocean. Five editions of the book were issued during the lifetime of the author. This, the sixth edition, has been edited and revised by his son with a commendable desire to keep green the memory of his illustrious father. We believe that he has succeeded in the aim that he modestly states to be to make this work still worthy of the favor that previous editions have enjoyed.

The fifth edition was issued in the autumn of 1892, just previous to the publication of the pharmacopeia of the United States. To make it correspond with the names officially recognised in the new pharmacopeia, the necessary additions and alterations have been instituted and several new illustrations have been added. In its present form the book shows abundant evidence of having kept pace with the progress of the subject with which it deals.

---

INDEX-CATALOGUE OF THE LIBRARY OF THE SURGEON-GENERAL'S OFFICE, United States Army. Authors and Subjects. Together with an Alphabetical List of Abbreviations of Titles employed in the Index-Catalogue. Volume XVI. W—Z Y T H U S. Washington: Government Printing Office. 1895.

This volume, the final one of the first series, includes 12,579 author-titles, representing 4,857 volumes and 11,613 pamphlets. It also contains 8,312 subject-titles of separate books and pamphlets and 13,280 titles of articles and periodicals. The entire series, sixteen volumes, 176,364 author-titles, representing 85,663 volumes and 151,504 pamphlets. They also contain 168,557 subject-titles of separate books and pamphlets, 511,112 journal articles and 4,335 portraits.

The editor announces that the manuscript of a second series including all the titles of books and articles received too late for the first series has been prepared and will, probably, make five printed volumes of the same size as those constituting the first series.

The foregoing statement indicates the great labor performed in the preparation of this catalogue, which ought to command the grateful appreciation of physicians all over the world.

---

THE URINE IN HEALTH AND DISEASE AND URINARY ANALYSIS, Physiologically and Pathologically Considered. By D. CAMPBELL BLACK, M. D., L. R. C. S., Professor of Physiology, Anderson College Medical School. In one 12mo volume of 256 pages, with seventy-three engravings. Cloth, $2.75. Philadelphia: Lea Brothers & Co., Publishers. 1895.

The importance of a thorough understanding of the part played by the kidney in the economy as an eliminator of waste products cannot be overestimated. The urine must be interpreted intelligently, else many of the phenomena of disease will be lost sight of. This little treatise cannot fail to aid the student in a better understanding of the subject. It is also a valuable book for the practising physician to consult in his urinary investigations. The physiological chemist, too, will find it of great value. It is concise and practical in its treatment of the subject.

---

THE JOHNS HOPKINS HOSPITAL REPORTS. Report in Gynecology, III., Volume IV., Number 7-8. Baltimore: The Johns Hopkins Press. 1895.

The excellent character of some of the work done at the Johns Hopkins medical school and hospital is testified to in this report, which appears to be an honest setting forth of cases that the author himself has seen and faithfully recorded. They cannot fail to interest every gynecologist. The pamphlet is illustrated by a number of fine lithographs executed in Leipzig. There are also two fine micro-photographs well illustrating tuberculosis of the endometrium.

